# Application of Computer Vision to the Automated Extraction of Metadata from Natural History Specimen Labels: A Case Study on Herbarium Specimens

**DOI:** 10.3390/plants15040637

**Published:** 2026-02-17

**Authors:** Jacopo Zacchigna, Weiwei Liu, Felice Andrea Pellegrino, Adriano Peron, Francesco Roma-Marzio, Lorenzo Peruzzi, Stefano Martellos

**Affiliations:** 1 Department of Mathematics, Informatics and Geosciences, University of Trieste, Via Weiss 2, 34127 Trieste, Italy; jacopo.zacchigna@studenti.units.it (J.Z.); adriano.peron@units.it (A.P.); 2 Department of Life Sciences, University of Trieste, Via Licio Giorgieri 10, 34127 Trieste, Italy; martelst@units.it; 3 Department of Engineering and Architecture, University of Trieste, Via Alfonso Valerio 6/1, 34127 Trieste, Italy; fapellegrino@units.it; 4 Botanic Garden and Museum, University Museum System, University of Pisa, Via Ghini 13, 56126 Pisa, Italy; francesco.romamarzio@unipi.it; 5 PLANTSEED Lab, Department of Biology, University of Pisa, Via Derna 1, 56126 Pisa, Italy; lorenzo.peruzzi@unipi.it; 6 Centro Interuniversitario per la Biodiversità Vegetale Big Data—PLANT DATA, Department of Biological, Geological and Environmental Sciences, Alma Mater Studiorum University of Bologna, Via Zamboni 33, 40126 Bologna, Italy

**Keywords:** artificialintelligence, biodiversity, digitization, multimodality, vision-language transformer

## Abstract

Metadata extraction from natural history collection labels is a pivotal task for the online publication of digitized specimens. However, given the scale of these collections—which are estimated to host more than 2 billion specimens worldwide, including ca. 400 million herbarium specimens—manual metadata extraction is an extremely time-consuming task. Thus, automated data extraction from digital images of specimens and their labels therefore is a promising application of state-of-the-art computer vision techniques. Extracting information from herbarium specimen labels normally involves three main steps: text segmentation, multilingual and handwriting recognition, and data parsing. The primary bottleneck in this workflow lies in the limitations of Optical Character Recognition (OCR) systems. This study explores how the general knowledge embedded in multimodal Transformer models can be transferred to the specific task of herbarium specimen label digitization. The final goal is to develop an easy-to-use, end-to-end solution to mitigate the limitations of classic OCR approaches while offering greater flexibility to adapt to different label formats. Donut-base, a pre-trained visual document understanding (VDU) transformer, was the base model selected for fine-tuning. A dataset from the University of Pisa served as a test bed. The initial attempt achieved an accuracy of 85%, measured using the Tree Edit Distance (TED), demonstrating the feasibility of fine-tuning for this task. Cases with low accuracies were also investigated to identify limitations of the approach. In particular, specimens with multiple labels, especially if combining handwritten and typewritten text, proved to be the most challenging. Strategies aimed at addressing these weaknesses are discussed.

## Simple Summary

Extracting metadata from natural history collection labels is pivotal for the online publication of digitized specimens. Building on a pre-trained multimodal Transformer, we developed an end-to-end automated solution to extract metadata from digitally imaged herbarium specimen labels and map them to Darwin Core standard concepts. A second objective was to demonstrate the feasibility of applying state-of-the-art AI techniques to biodiversity data through a real-world use case that does not require image preprocessing or additional manual labeling for training. The proposed solution does not rely on closed-source services, is fine-tuned in-house, and can be used offline and locally. It can be flexibly reused by developers to extract metadata across different herbarium collections. Furthermore, its encoder and/or decoder component can be replaced to take advantage of newer foundational models as soon as they become available. This approach is also particularly well suited to ensuring data privacy. Finally, the publication of our baseline model could serve as a benchmark for comparison with different solutions that aim for greater generality and accuracy.

## 1. Introduction

Natural history collections (NHCs) host large amounts of specimens collected since the 17th century. The total number of specimens worldwide is estimated to exceed 2 billion [1]. Herbaria, which are natural history collections of dried plant specimens, are estimated to contain ca. 400 million specimens globally [2]. Each specimen tipically has one or more labels reporting metadata such as the scientific name, date and locality of collection, collector, and other relevant information. These data are pivotal for understanding the evolution of biodiversity [3], forecasting its future changes, and supporting a wide range of other research topics [4,5].

The mobilization of specimen data through digitization is therefore particularly relevant [6,7,8]. While many digitization efforts are carried out manually, large-scale and industrialized workflows aimed at improving efficiency have been developed for botanical collections [9] and, more recently, for entomological collections. In general, contemporary digitization efforts follow an image-to-data workflow [10], in which digital images of specimens and their labels are captured and metadata are subsequently transcribed. This approach minimizes physical handling of specimens, which, as biological objects, are particularly susceptible to deterioration [11,12]. Label data are then typically published in “global” repositories such as the Global Biodiversity Information Facility (GBIF), using common standards such as Darwin Core [13].

To further increase the efficiency of digitization efforts, automated metadata extraction using computer vision is being actively investigated [14,15,16,17], as manual metadata extraction is highly labor-intensive [18,19]. Early approaches to automated extraction largely relied on standard Optical Character Recognition (OCR) as a primary means for recognizing printed and handwritten text on labels. Consequently, most previous approaches combined OCR with Natural Language Processing (NLP) techniques. OCR was used to extract unstructured text from images, while NLP techniques addressed tasks such as Name Entity Recognition (NER). These systems typically provide interfaces that allow human operators to correct errors or enter missing data. HERBIS19 [20] and SALIX20 [14] are representative examples of this design paradigm. While OCR-based approaches can speed-up data entry by supporting label transcription, they remain limited by poor label quality and other sources of noise [14]. Thus, they still require substantial human oversight in day-to-day operations, as their reliability remains limited at the current state of the art. More recently, advances in OCR technology and the emergence of Large Language Models (LLMs) have enabled the development of new integrated workflows. Systems such as “Publish First” [21] and Hespi [22] are examples of this newer approach. However, these solutions typically rely on fully developed services accessed through external APIs, often managed commercial entities. This introduces several drawbacks, among which the limited transparency and controllability of workflow components that depend on external services. Except for reporting satisfactory results obtained using a pipeline that incorporate tools such as GPT-4, it is often not possible to provide detailed justifications for service selection or thorough analyses of failure cases [21]. Furthermore, data privacy and confidentiality cannot always be adequately guaranteed when using LLMs accessed via APIs, as such services may store or misuse sensitive information, thus exposing users to the risk of data leakage [23].

A fully trainable and configurable information extraction (IE) model based entirely on open-source solutions is, however, still lacking. This challenge has been partly addressed by the development of an automated label data extraction and database generation system from herbarium specimen images using OCR and NER techniques [24]. In this workflow text is first extracted using the Google Cloud Vision OCR service and subsequently parsed with the SpaCy python package for NER. While this NER-based parsing approach is both promising and innovative, the OCR component lacks the flexibility required to handle the complexity of heterogeneous label formats. Conventional OCR systems can detect and recognize textual regions in a document, but they do not interpret the semantic meaning of the text or its relationship to the spatial layout within the image. Furthermore, Google’s OCR is a closed-source service, thus limiting users ability to customize or adapt it to specific needs. With the advent of new techniques in NLP and Computer Vision (CV), driven by the rise of Transformer architectures [25], alternative approaches that leverage their power and versatility have become feasible. Through Transfer Learning [26], Large Vision-Language Models (LVLMs), pre-trained on large sets of general data, can be fine-tuned for domain-specific tasks using relatively limited training data. Since the introduction of the Multimodal Transformer (MulT) [27], which employs cross-modal attention mechanisms to reduce the need for explicit data alignment, numerous language–vision Transformer models have been developed. These approaches have demonstrated remarkable performances, as shown by TrOCR [28] and the Document Understanding Transformer (Donut) [29]. Language-vision models typically consist of an image encoder and a text decoder and are trained to both recognize texts in images and extract structured information, albeit with come differences. TrOCR processes text lines as input and is therefore not suitable for NER out of the box, whereas Donut is inhenrently better suited for infromation extraction tasks [28].

This research aims at describing the fine-tuning of Donut [29] for the automatic extraction of information from herbarium specimen labels and its mapping to the concepts of the metadata standard scheme Darwin Core [13], which is internationally adopted to ensure interoperability. To the best of our knowledge, this work is the first real-world application of state-of-the-art multimodal Transformer to support the challenging tasks of herbarium specimens digitization.

## 2. Results and Discussion

Three fine-tuning experiments were conducted using different image resolutions, and the performance of all models was evaluated on the same test dataset at the corresponding resolutions: 600 × 800, 960 × 1280, and 1200 × 1600. As shown in Table 1, TED accuracies increase with image resolution. Consequently, the 1200 × 1600 resolution was selected for subsequent inference, as it achieved the best TED accuracy median score.

To assess whether the pre-trained Donut model effectively transferred learned representations to the IE task of specimen labels processing, we also trained the model from scratch using images at 1200 × 1600 and the same hyperparameters employed during fine-tuning, except the disabled early stopping strategy. Training from scratch probably experience more bumpy fluctuations than fine-tuning. It can be stopped at initial epochs with the same “patience” parameter of early stopping strategy. For comparability, the scratch training was limited to five epochs, matching the number of epoch used in fine-tuning with 1200 × 1600 images.

**Figure 1:**
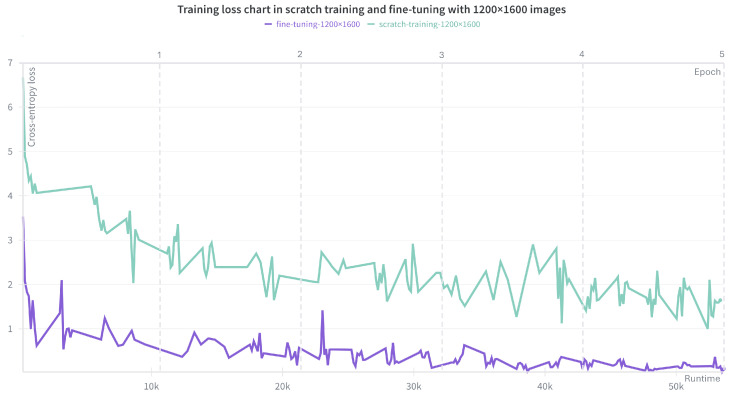
Training loss curves for training from scratch (green) and fine-tuning (purple) using images at a resolution of 1200 × 1600 over 5 epochs. The curves represent the decrease in cross-entropy loss during training. Even with extended training, the loss for training from scratch remain substantially higher, indicating inferior performance.

After five epochs of training from scratch without early stopping, only limited improvements were observed in both training loss and evaluation score. As shown in Figure 1, the loss curves for training from scratch remain substantially higher than those obtained through fine-tuning. A detailed quantitative comparison is provided in Table 2.

**Figure 2:**
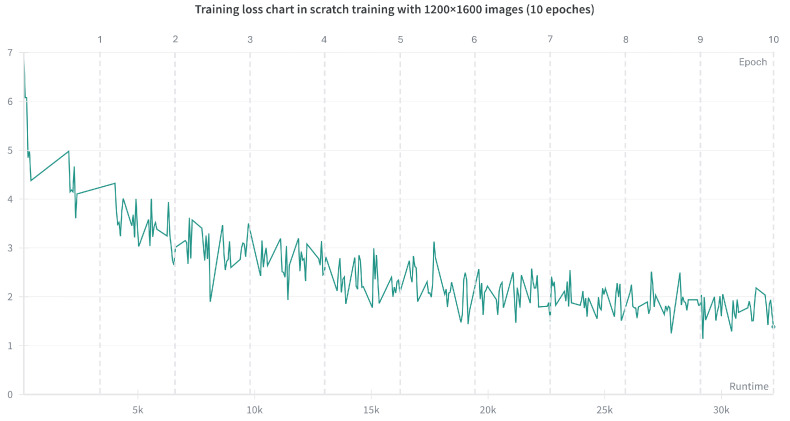
Training loss curves for training from scratch using images at a resolution of 1200 × 1600 over 10 epochs. The curves show the evolution of cross-entropy loss during training. Compared with the setup in Figure 1, this experiment used four A100 GPUs, which substantially reduced runtime despite the increased number of epochs. Nevertheless, the training loss remained high, underscoring the limitations of training from scratch using a limited dataset.

To exclude the possibility that the inferior performance of training from scratch was due to insufficient optimization, we extended the training to 10 epochs using the Adam optimizer [30] with a scheduled learning rate and an initial value of 1 × $10^{-5}$. Early stopping was disabled to ensure adequate training. As shown in Figure 2, the training loss decreased only gradually and remained around 2 for more than five epochs. This result underscores the limitations of training from scratch when only a limited amount of training data is available.

**Figure 3:**
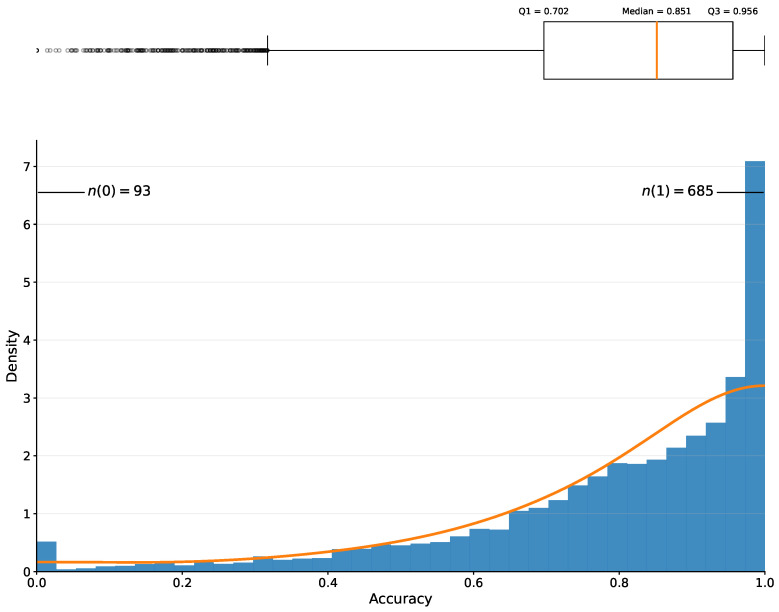
Boxplot (top) and histogram (bottom) of accuracy scores on the test set using 1200 × 1600 images. The 25th (Q1) and 75th (Q3) percentiles are 0.702 and 0.956, respectively. Median and mean values are reported in Table 1 and Table 2. Over 75% of test images achieve an accuracy score of more than 0.7, while low-accuracy cases are rare. In the histogram, the frequencies at accuracy scores of 0 and 1 are indicated as n(0) and n(1), since the kernel density estimation curve (orange) smooths out these peaks. Among 6895 test images, n(0) and n(1) account for 1.3% and 9.9%, respectively.

As shown in Table 1 and Table 2, although the median and mean TED accuracy are not optimally close the maximum possible score of 1, the evaluation results are satisfactory given the overall distribution of TED accuracy in the test dataset. Over 75% of test cases achieved a TED accuracy greater than 0.702 (Figure 3), and the top 25% exceed 0.956.

**Figure A1:**
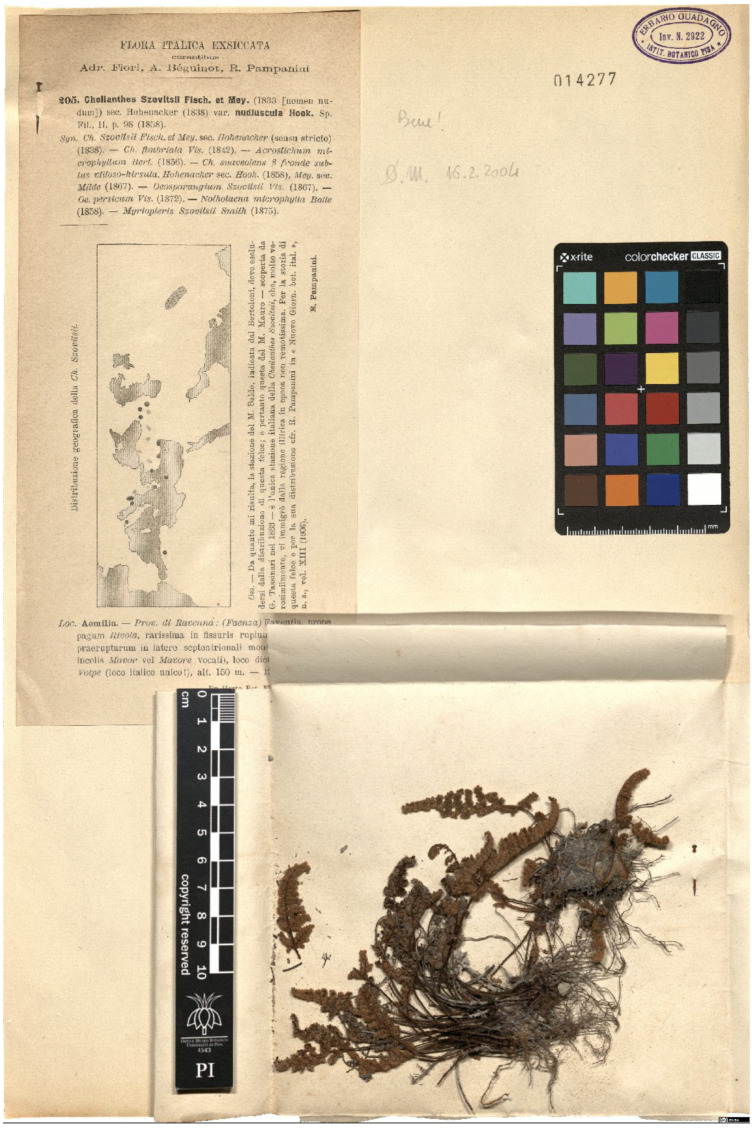
Upper image, Table 3.

**Figure A2:**
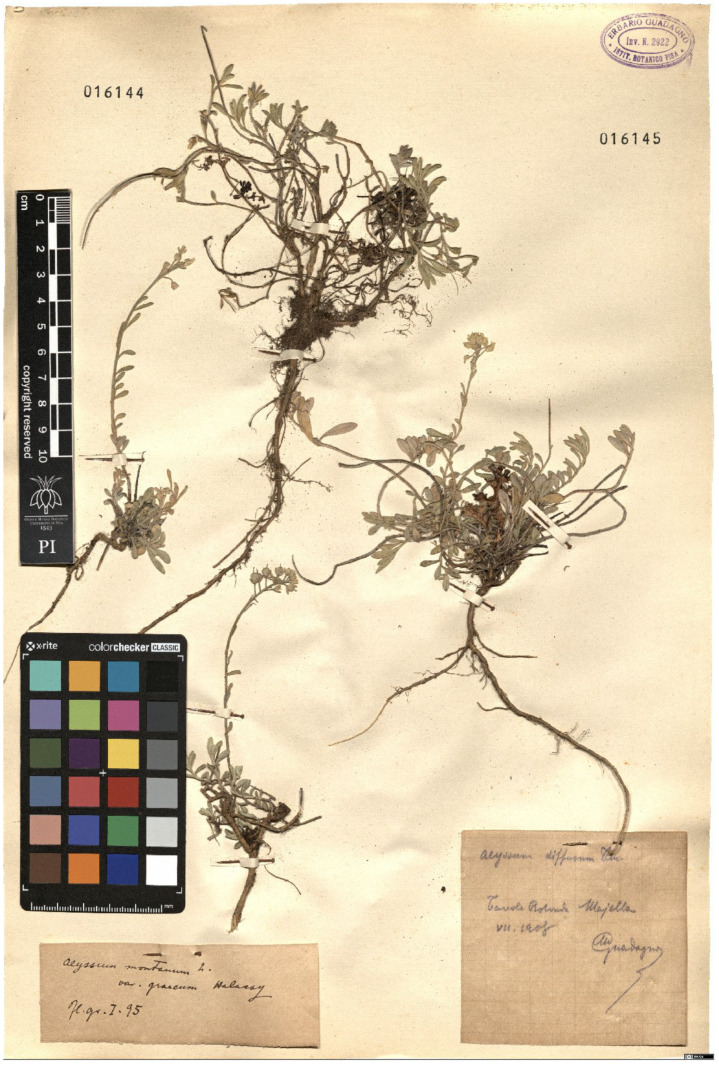
Lower image, Table 3.

**Figure A3:**
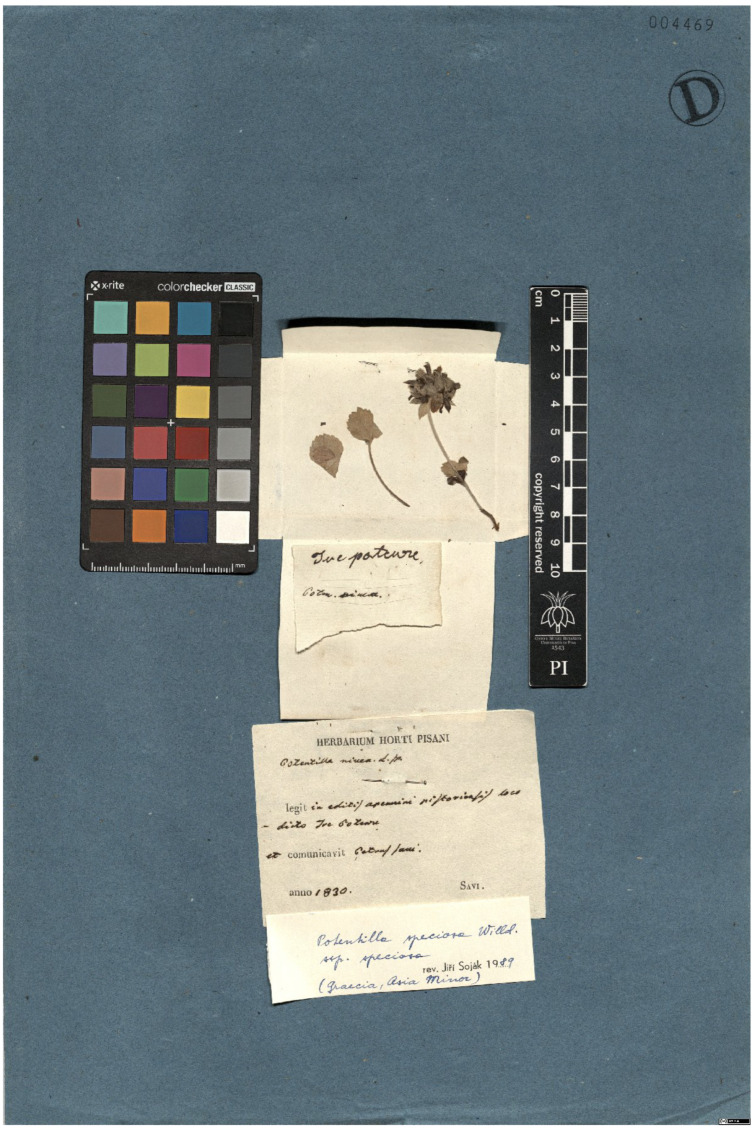
Upper image, Table 4.

**Figure A4:**
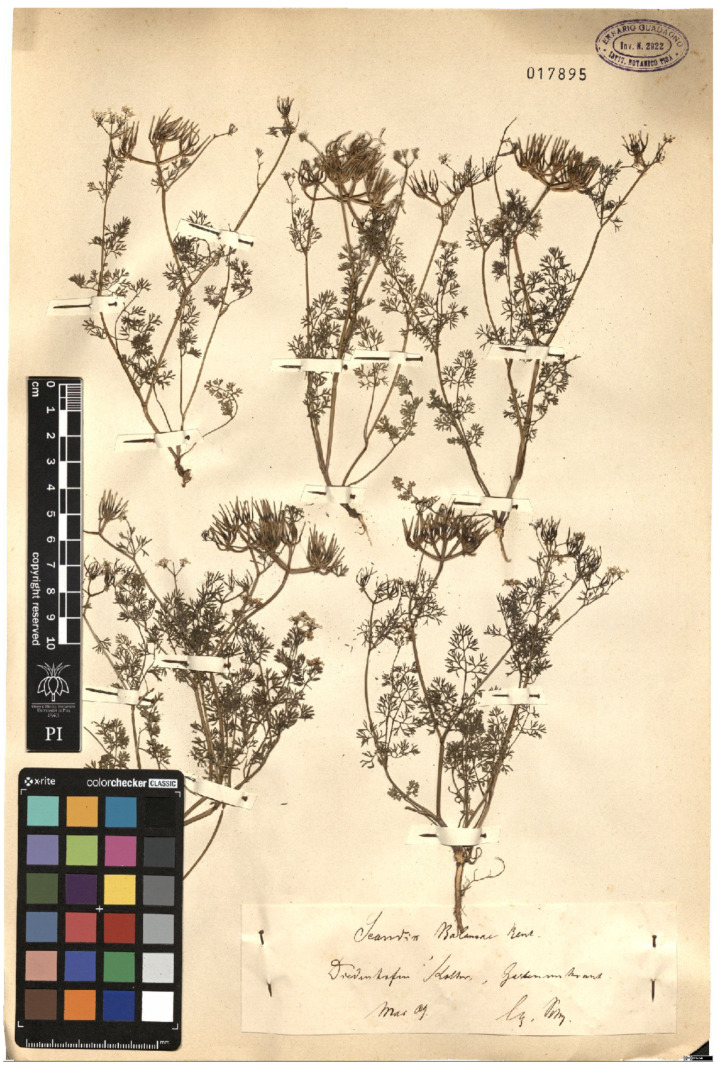
Lower image, Table 4.

**Figure A5:**
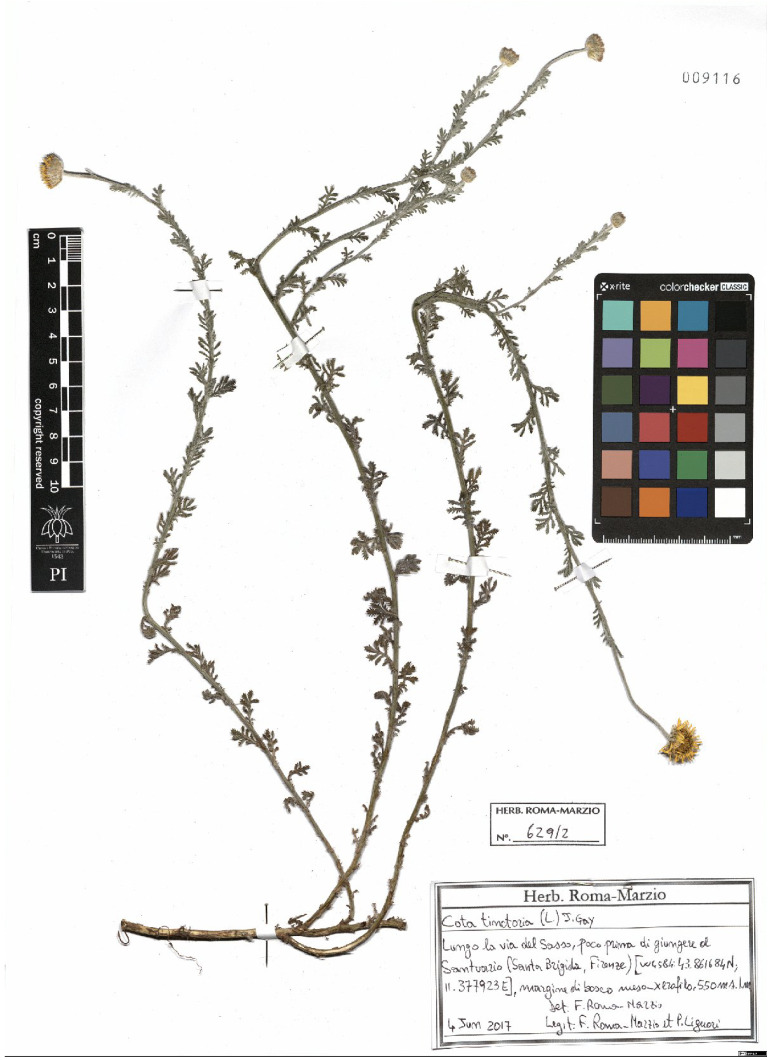
Upper image, Table 5.

**Figure A6:**
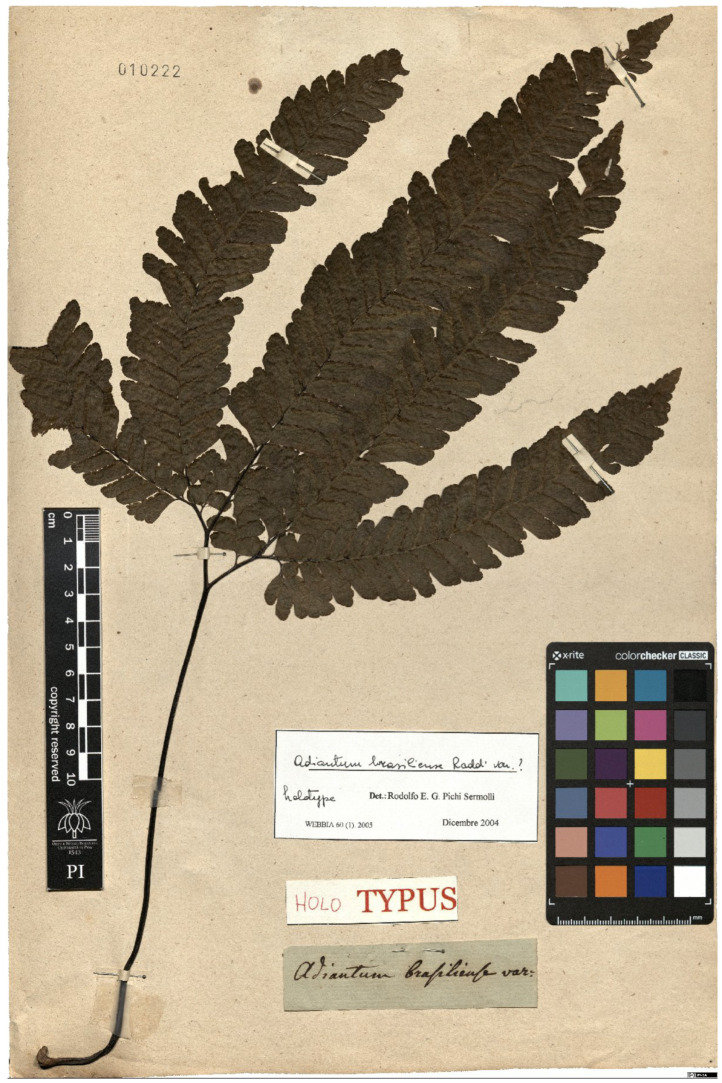
Lower image, Table 5.

**Figure A7:**
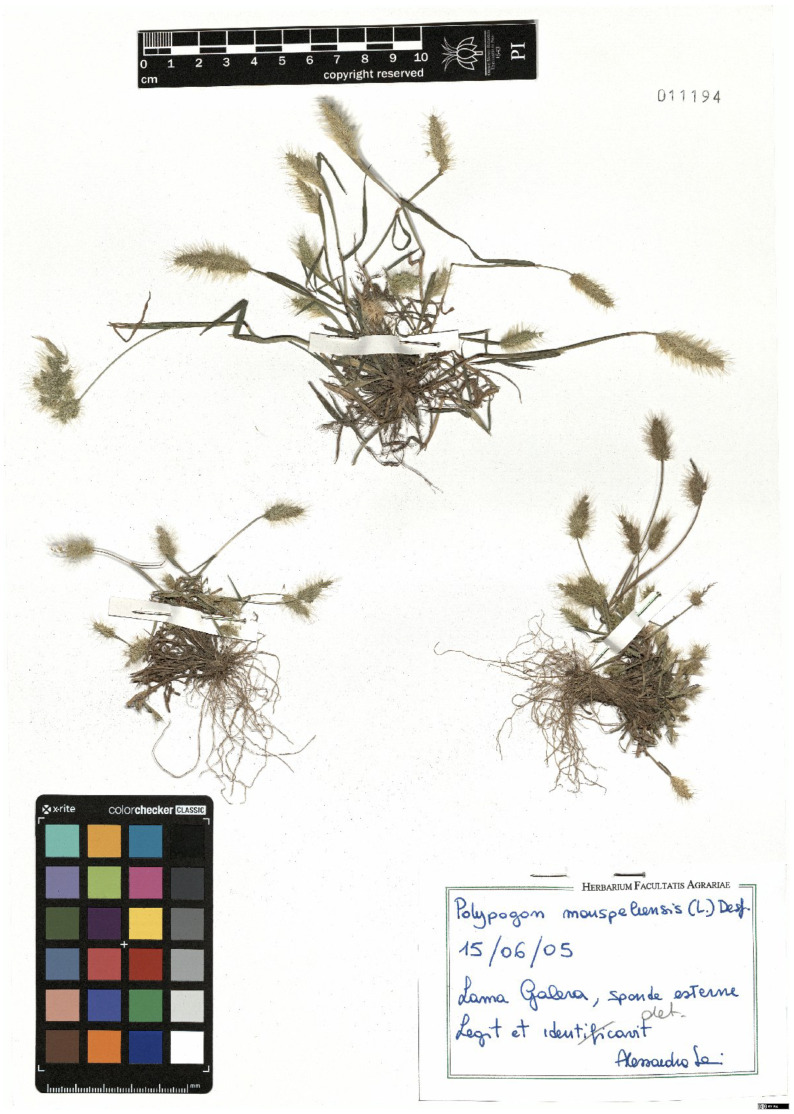
Upper image, Table 6.

**Figure A8:**
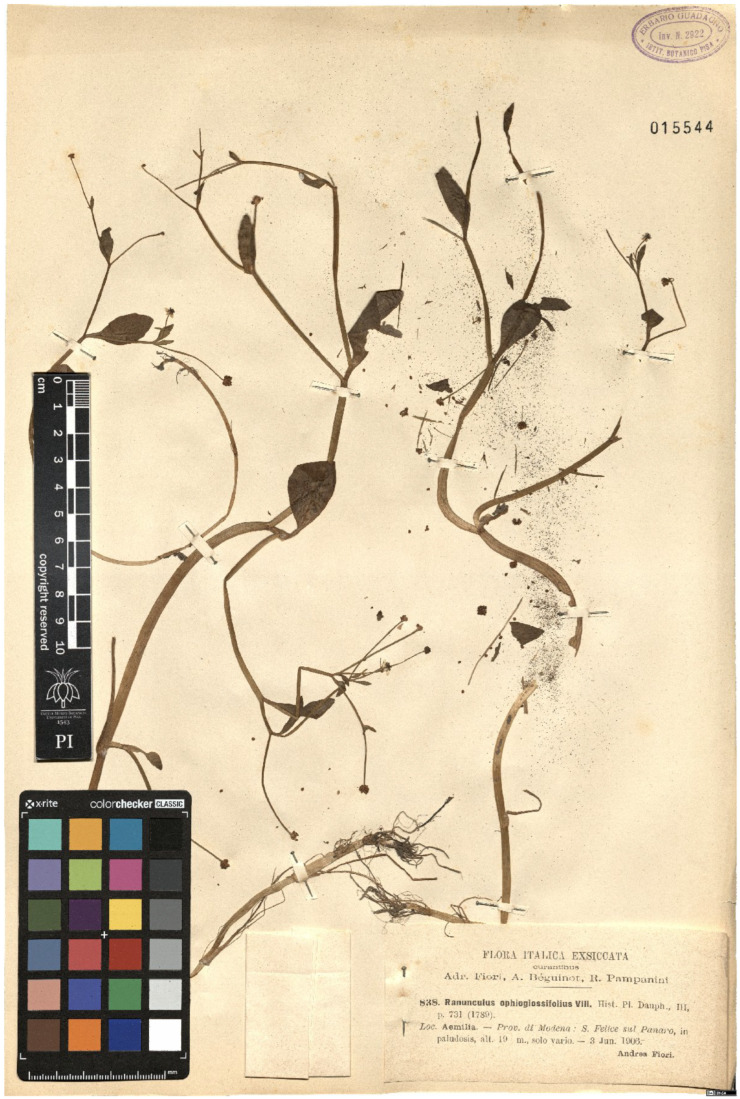
Lower image, Table 6.

Lower performance scores not necessarily indicate errors in text identification. In several cases, they represent “false negatives”, often arising from discrepancies between the ground truth and the text actually present on the specimen labels. Table 3, Table 4, Table 5 and Table 6 showcase representative examples spanning the full range of performance, from the lowest to the highest accuracy scores. The corresponding images are also provided as Appendix A (Figure A1, Figure A2, Figure A3, Figure A4, Figure A5, Figure A6, Figure A7 and Figure A8). In the tables, text shown in green indicates portions that are identical between the ground truth and the model prediction, while text shown in red indicates discrepancies.

Table 3 exhibit two specimens with a TED accuracy score of 0. In the first case, HeR-T correctly extracted the scientific name, albeit partially, as it failed to extract authorities and varietal information. Notably, the ground truth for this specimen does not reflect the text on the label. Instead, it was updated to account for recent nomenclatural changes: one of the synonyms listed on the label corresponds to the currently accepted taxon name, whereas the name originally used on the label is a *nomen nudum* (i.e., not validly published), and is therefore no longer considered valid.

The extracted gathering locality in this case is also partially correct; however, subsequent Latin text (partly obscured in the image by specimen envelope) was not accurately extracted. Although the overall TED accuracy score for this specimen is 0, the model suiccesfully extracted meaningful portions of the label content. This case can therefore be classified as “false negatives” resulting from discrepancy between the model outputs and the human-curated transcriptions.

In the second case presented in Table 3, the TED accuracy of 0 arises from the presence of two specimens mounted on a single sheet, while the ground truth includes the transcription only of the first specimen. HeR-T, by contrast, (correctly) extracted metadata from the second label only. Had this extraction been used as the reference, the resulting TED accuracy would have been close to 1. However, the model failed to extract information from both labels.

Together, these two edge cases highlight the challenges faced by HeR-T when processing specimens with complex layout and ambiguous definitions of metadata fields.

Table 4 showcases two examples of poor performance (TED accuracy score of ca. 0.2). In the first case, the lower TED accuracy is likely due to the presence of three labels on a single specimen sheet, rather than the more typical single label. HeR-T extracted the scientific name (although missing the varietal epithet) from the bottom label, while the gathering event date was taken from the second label. Other information, such as the gathering locality, were not extracted. The ground truth, however, does not correspond to a single label. Instead, the scientific name was taken from the third label and is itself incorrectly formatted, as the authority (Willd.) should follow the binomial (*Potentilla speciosa*), rather than the varietal epithet (var. *speciosa*). Similarly, the ground truth combines the gathering event date from the second label with the locality, further complicating the evaluation.

In the second case, HeR-T extracted a scientific name that is correct at the genus level (*Scandix*), but the species epithet and authority are incorrect. The gathering event locality was fully extracted, whereas it was only partially transcribed in the ground truth. This discrepancy suggests that HeR-T may have misinterpreted the text which was not included in the ground truth transcription. The gathering event date, however, was correctly identified.

Table 5 reports are two examples in which the TED accuracy score exceeds 0.8. In both cases, the discrepancies between the predicted text and the ground truth are minimal and largely negligible. In the first example, HeR-T perfectly predicted the label content; the only difference concerns the inclusion of the altitude within the locality field. Since the altitude is repeated in the correct field as well, this discrepancy reflects an issue in output organization rather than a recognition error.

The second case is particularly noteworthy. Although the TED accuracy score is lower than 1, the discrepancy arises from a typo in the ground truth. Donut obviously did not reproduce the typo (text in red in the grounfd truth in the second example of Table 5). It should however be noted that for this specimen both the ground truth and the model transcription introduce an error in the scientific name. Specifically, the authority (Raddi) is misinterpreted as the varietal epithet. On the label the scientific name is correctly reported as *Adiantum brasiliense* Raddi and is followed by the string “var.?”, which indicates that the specimen is likely a variety of the nominal species, although the variety itself has not yet been identified. In contrast, both the ground truth and the model transcription incorrectly place the authority (Raddi) after the “var.” string.

Table 6 displays examples achieving a perfect TED accuracy score (1.0). In both cases, the predicted text exactly matches the ground truth, which is an accurate transcription of the labels content. While the first specimen has a handwritten label and the second a typewritten one, both sheets have a single label and a single specimen, an ideal scenario for accurate transcription.

## 3. Materials and Methods

### 3.1. Dataset

This study was carried out using a dataset of digitized specimens from the Herbarium of the University of Pisa (international acronym PI). The dataset comprises 55,089 specimens that have been digitized and published online through the JACQ Virtual Herbaria platform (http://www.jacq.org/), a continuously evolving consortium of virtual herbaria based in Vienna [31,32]. The collection includes specimens gathered from 97 countries and spans a temporal range of approximately two centuries. A large portion of the specimens derives from a recently completed digitization project focused on the Herbarium Guadagno [33].

Specimen images in the dataset are provided in JPEG format, while the associated metadata—derived from manual transcription of the original specimen labels—are organized in a spreadsheet in which each row is a specimen. After removing images lacking corresponding metadata entries, the final dataset comprised 45,951 specimens.

For fine-tuning, a dedicated dataset was created by pairing each specimen image with a JSONL (https://jsonlines.org/) ground truth, consisting of four essential metadata fields. These fields represent the core information typically associated to a published specimen record according to the Darwin Core standard: original scientific name, collection date, collection locality, and elevation.

It should be noted that not all fields present in the transcribed metadata spreadsheet were included in the fine-tuning dataset. This decision reflects the fact that the metadata in the original dataset do not always correspond to the text appearing on the specimen labels. Such discrepancies are primarly due to updates in scientific nomenclature (i.e., when an original name has been replaced with a currently accepted name without modifying the original label), or to the partial transcription of other label information.

Ideally, fine-tuning would rely exclusively on specimen images whose ground truth data exactly match the text on labels. However, in practical biodiversity research workflows, minor interpretative steps during metadata transcription are common and often unavoidable. This situation introduces a trade-off between strict textual alignment and adherence to real-world transcription practices. Consequently, we prioritized metadata fields with clear definitions, minimal semantic ambiguity, and relatively few missing values, while excluding fields that require extensive interpretation or post-processing.

As an initial benchmarking step towards an end-to-end solution for automated metadata extraction, the selected subset of Darwin Core fields—chosen to minimize the noise introduced by transcription-related interpretation—was deemed sufficient to evaluate the feasibility and effectiveness of the proposed fine-tuning approach.

### 3.2. Base Model Selection

Given the limited size of the dataset, rather than training a model from scratch, we employed a transfer learning approach known as fine-tuning. This enabled us to adapt a model to a specific and narrow domain with minimal additional training data. Accordingly, a base model pre-trained on a large-scale dataset for general knowledge has been selected. Candidate base models were required to support multiple languages and complex label formats, without being constrained by OCR limitations. Performance on widely used CORD benchmark [34], which consists of 10000 Latin alphabet receipt images, was chosen as a suitable criterion for comparing the capabilities of different base models (see Table 7).

Donut was selected as the base model for fine-tuning. While BERT can serve as a baseline for any IE task doe to its adaptability to multiple languages, it lacks the ability to recognize texts directly from images and therefore must rely on an external OCR engine. In contrast, Donut was specifically designed to recognize text within images, making it a more suitable choice. TrOCR also employs an encoder-decoder architecture similar to Donut, but it requires “text-line” images as input [28]. These images must be produced through a preprocessing segmentation step, in which the original image is cropped into separate portions, each containing a single line of text. Consequently, TrOCR can extract text from images but is not suitable to parse complex document structures. Donut, by contrast, is an OCR-free model capable of extracting information directly from documents without any preprocessing. Furthermore, it is trained on a multilingual dataset that includes Chinese, Japanese, Korean, and English.

### 3.3. Dataset Preprocessing

In our proposed multimodal Transformer, the pre-trained image encoder and text decoder were fine-tuned using pairs of specimen images and their corresponding ground truth annotations. The following data preparation steps were applied prior to fine-tuning:

1. Image resolution was reduced to limit computational requirements, as well as to evaluate its impact on model performance. Three datasets were prepared with images at different resolutions (600 × 800, 960 × 1280, and 1200 × 1600 pixels) to assess the IE capabilities of the model.
2. The ground truth was organized in JSONL format, with keys represented by special tokens that had been previously added to the model’s tokenizer, following the approach described in [29].

Furthermore, during inference with the pre-trained model, the text decoder generates output beginning from the standard start token “<s>”. For the fine-tuned model, a task-specific prompt token “<s_herbarium>” was added as the start of the sequence, replacing the general start token “<s>” during inference.

### 3.4. Experiment Environment Setting Up

Donut was fine-tuned on the CINECA (https://www.cineca.it/en, accessed on 12 February 2026) HPC facility, specifically using the Leonardo Booster module. The environment was a single compute node equipped with one Intel Xeon 8358 CPU with 32 cores running at 2.6 GHz, four NVIDIA Ampere A100 GPUs with 64GB of memory each, and 512 GB RAM. The batch size across fine-tuning experiments depending on image resolution (See Table 8). All other hyperparameters, including precision, optimizer, and learning rate, followed the recommendations provided in the Donut paper [29]. The code for the fine-tuned model (named HeR-T—Herbarium label Recognition-Transformer), along with detailed settings, is available on GitHub, updated on 25 January 2026: https://github.com/elderprince/HeR-T-Fine-tuning, accessed on 12 February 2026.

### 3.5. Fine-Tuning

Fine-tuning is an effective strategy when the target dataset for re-training is significantly smaller than the dataset used for the pre-training [39]. The selected base model, Donut, was pre-trained on 2 million synthetically generated images, which is considerably larger than our target dataset of 45,951 specimen images.

**Figure 4:**
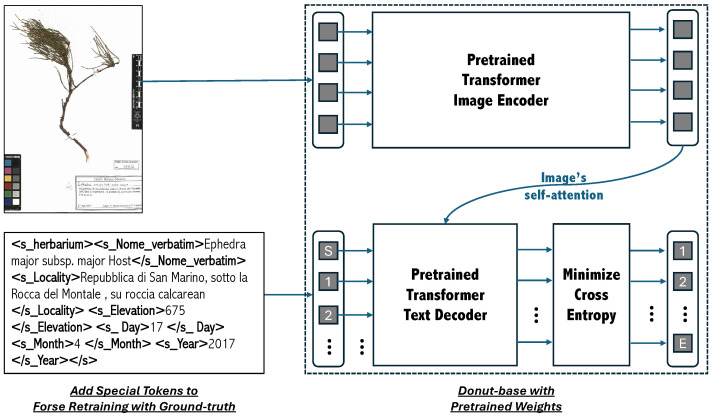
Fine-tuning workflow with a set of pairs of specimen image/ground truth. Images are converted in RGB and fed to the pre-trained encoder for self-attention. The encoder output is then passed to the pre-trained decoder for cross-attention, aligning the image features with the parsed ground truth. Fine-tuning minimizes the cross-entropy loss function, guiding the model to generate output that match the ground truth.

Furthermore, when the base task and target task are similar, the transferability of learned features can enhance the efficiency of fine-tuning [39]. In our case (see Figure 4), the target task—IE from specimen labels—can be seen as a sub-task of Donut’s broader text recognition task. As a result, the features learned by Donut are largely transferable, enabling effective fine-tuning. In this research, we performed a full fine-tuning without freezing any layers.

Cross-entropy was used as the loss function for fine-tuning. Each token prediction is treated as a classification problem, conditioned on both the image and the context provided by previously generated tokens [29].

However, Large Vision-Language Models (LVLM) such as Donut are prone to overfitting when the target dataset for fine-tuning is relatively small [39]. To prevent overfitting, early stopping was implemented during fine-tuning. Specifically, the process was halted (early stopping) whenever the Tree Edit Distance (TED) accuracy on the validation set did not improve over the previous evaluation. During fine-tuning, TED accuracy (see next section) on the validation set was evaluated every 25% of an epoch was (i.e., four evaluations per epoch). To account for performance fluctuations and avoid premature stopping, a “patience” parameter of 7 was introduced, allowing the model to continue for up to seven consecutive evaluations without improvement before halting.

### 3.6. Evaluation: TED Accuracy

Following the evaluation protocol abopted by [29], a TED [40] based score was used, as defined by the formula:

(1) max(0,1−TED(pr,gt)/TED(φ,gt),

where gt, pr, and φ stand for ground truth, prediction, and empty trees, respectively [29].

For brevity, we refer to the score defined in (1) as TED accuracy. Given the tree structures representing predicted text sequences, TED accuracy is computed using the Tree Edit Distance between the prediction and the ground truth, denoted as TED(pr, gt), and between the ground truth and the empty tree, denoted as TED (φ, gt). The fewer edits required to transform the prediction into the ground truth, the closer the TED accuracy to 1. Compared with traditional metrics such as the F1 score [41], which consider only word-level overlap between the prediction and the ground truth, TED accuracy also accounts for the structural organization of the predicted text sequence. This evaluation formulation was adopted by [29] and has been used in other IE approaches [35,42]. Accordingly, we employed TED accuracy both as a benchmarking metric and as the evaluation metric for the early stopping during fine-tuning.

## 4. Conclusions

Extracting metadata from natural history collection specimens is a challenging and labor-intensive task when performed manually [18,19]. Consequently, substantial effort has been devoted to the development of automated or semi-automated solutions. Most existing approaches rely on OCR pipelines, sometimes coupled with NLP or large language models, to support metadata extraction workflows [14,15,16,17,20,21,24]. While effective in specific contexts, many of these solutions depend on externally managed APIs, limiting customization, fine-tuning, and full end-to-end control over the extraction process.

In this study, we explored a novel approach based on Transfer Learning [26], which enables fine-tuning of Large Vision Language Models (LVLMs) pre-trained on extensive general-purpose datasets. Specifically, we adopted Donut [29] as the base model. Compared to training a model from scratch, fine-tuning a pre-trained LVLM resulted in substantial performance gains. Training from scratch was constrained by the limited size of the available dataset and yielded markedly lower performance. In contrast, fine-tuning required less training time and achieved markedly better results, with a TED of 0.910 compared to 0.629 in the best-case training from scratch scenario.

Beyond dataset size, training performance is strongly influenced by data quality. Domain-specific datasets derived from digitized natural history collections do not always preserve the original ground truth as it appears on specimen labels; instead, they often contain curated or standardized metadata derived from the original text. As an example, locality information may be homogenized using gazetteers or toponymic database, rather than transcribed verbatim. Similarly, scientific names may be corrected, expanded (e.g., adding authorities), or updated to reflect current nomenclature without preserving the original label content. Such issues were present in the dataset used for the fine-tuning of Donut in this study.

Despite these limitations, fine tuning yielded overall satisfactory results (Figure 3). The median testing TED accuracy (0.851) indicates that the model predictions are generally close to the ground truth. As shown in Table 3 and Table 4, lower TED accuracy scores are not always due to prediction errors, but to discrepancies between the ground truth and the texts on the specimen labels, resulting in “false negatives”. Poorer performance is typically associated with (a) the presence of multiple labels on a single herbarium sheet, often corresponding to multiple specimen, (b) illegible handwriting, and/or (c) the use of elaborated or standardized ground truth during manual transcription.

In our results, specimens with a single label and clear handwriting (or typewritten labels) are generally predicted correctly. This scenario is common in modern collections, where herbarium sheets containing multiple labels are rare. Discrepancies introduced by transcription elaboration remain present; however, since such cases were minimized through field selection prior to fine-tuning, noise in the training of HeR-T can be at least partially controlled. Accordingly, the reported TED accuracy should be interpreted as a conservative baseline under realistic annotation conditions, rather than an upper bound of the model’s potential performance. These findings suggest that the proposed approach can be effectively integrated into herbarium digitization workflows.

The results of this study open several directions for future research. First, as additional metadata fields (such as collector information) become consistently available in new datasets, the JSONL ground truth schema can be expanded, enabling fine-tuning of an updated HeR-T for broader metadata extraction. Second, extracting illegible handwriting remains challenging, likely due to the limited representation of handwritten data in Donut’s pre-training. Incorporating both printed and handwritten samples during training could increase data diversity and improve generalization, thereby enhancing the replicability of this approach across different natural history collections. Third, for metadata fields which are uncommon or lack a consistent structure across specimens, such as habitat notes, the proposed method is not well suited for automated extraction. In such cases, research on label segmentation and classification could support the identification and exclusion of these fields for specialized downstream processing.

Finally, although Donut was used as the base model in this study, the same fine-tuning strategy can be applied to other general-purpose LVLMs. Given the rapid evolution of LVLM architectures, newer models could outperform Donut in this domain, offering further opportunities to improve automated metadata extraction from natural history collections.

## Figures and Tables

**Table 1:** Runtime and testing TED accuracy of three Donut fine-tuning configurations using image resolutions of 600 × 800, 960 × 1280, and 1200 × 1600. All models were fine-tuned on the same Leonardo HPC system equipped with NVIDIA Ampere A100-64 GPU to ensure comparable runtimes. TED accuracy was evaluated on the same held-out test dataset for all configurations.

	Runtime (s)	Average Testing TED Accuracy	Median Testing TED Accuracy	Standard Deviation of Testing TED Accuracy
600 × 800	38,715	0.607	0.629	0.254
960 × 1280	59,027	0.750	0.806	0.227
1200 × 1600	55,119	0.788	0.851	0.218

**Table 2:** Runtime and TED accuracies for training from scratch and fine-tuning using images at a resolution of 1200 × 1600. Both approaches were evaluated using the same test dataset and identical hyperparameters settings, except the disable early stopping strategy.

	Runtime (s)	Validation TED Accuracy	Average Testing TED Accuracy	Median Testing TED Accuracy	Standard Deviation of Testing TED Accuracy
Scratch Training	54,729	0.697	0.181	0.202	0.110
Fine-tuning	55,119	0.929	0.788	0.851	0.218

**Table 3:** Recognition examples with zero TED accuracy from the fine-tuned model using 1200 × 1600 images. Complex labels with extensive additional information, biased ground truth annotations, and images containing multiple specimens yield the lowest TED accuracy scores. In several cases, however, these results can be interpreted as “false negatives” (see text). Text shown in red indicates discrepancies between the ground truth and the prediction, while text shown in green denotes matching content.

Specimen (Figure A1 and Figure A2)	Ground Truth	Recognition	Accuracy Score
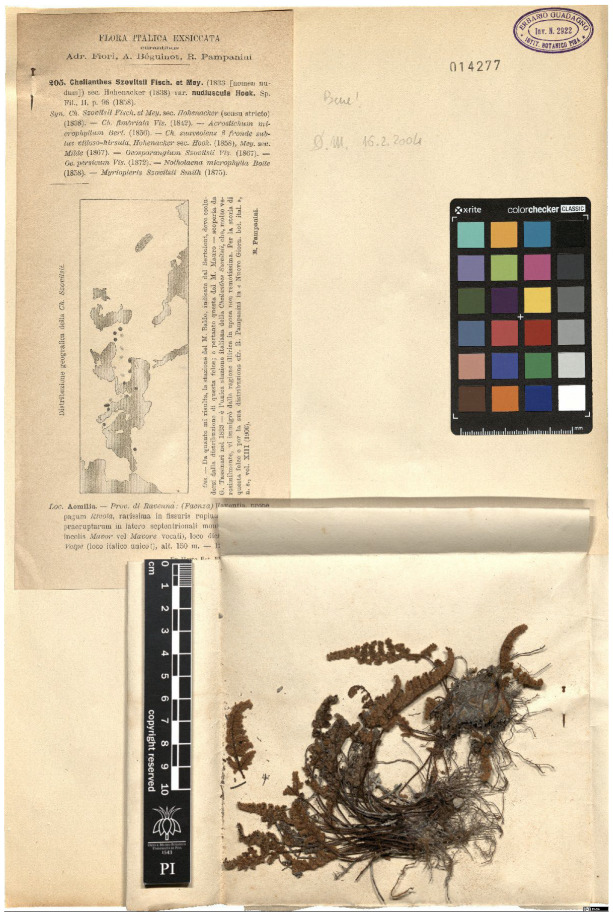	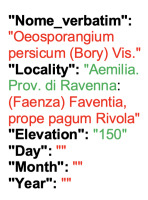	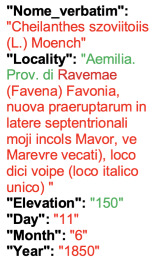	0
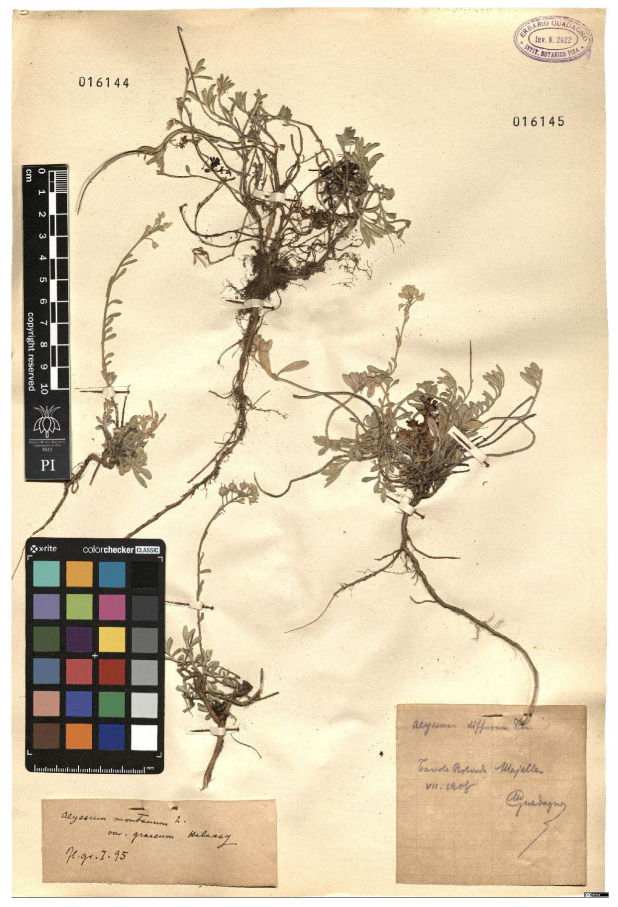	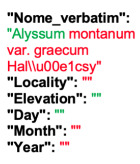	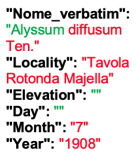	0

**Table 4:** Recognition examples with low TED accuracy from the fine-tuned model using 1200 × 1600 images. Images containing multiple labels and irregular handwriting can lead to reduced TED accuracy scores. Text shown in red indicates discrepancies between the ground truth and the prediction, while text shown in green denotes matching content.

Specimen (Figure A3 and Figure A4)	Ground Truth	Recognition	Accuracy Score
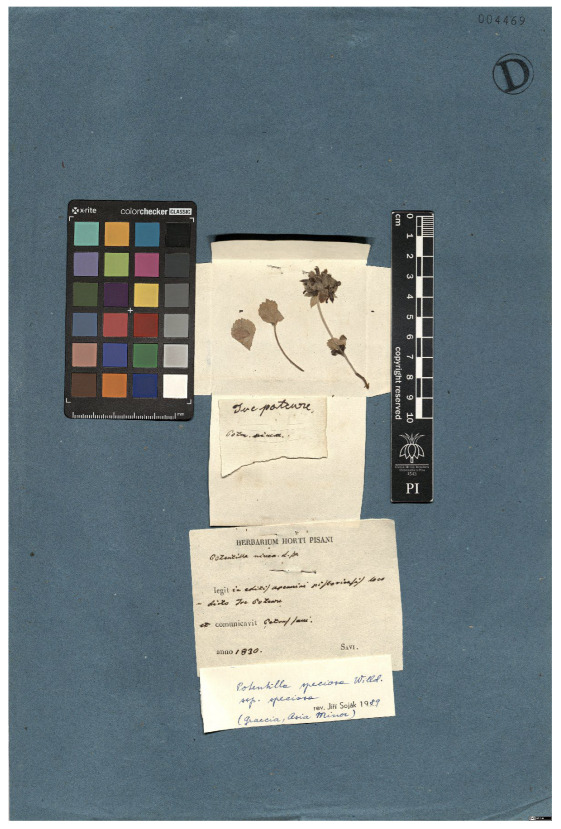	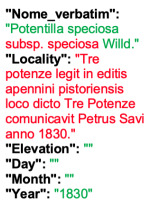	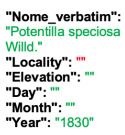	0.204
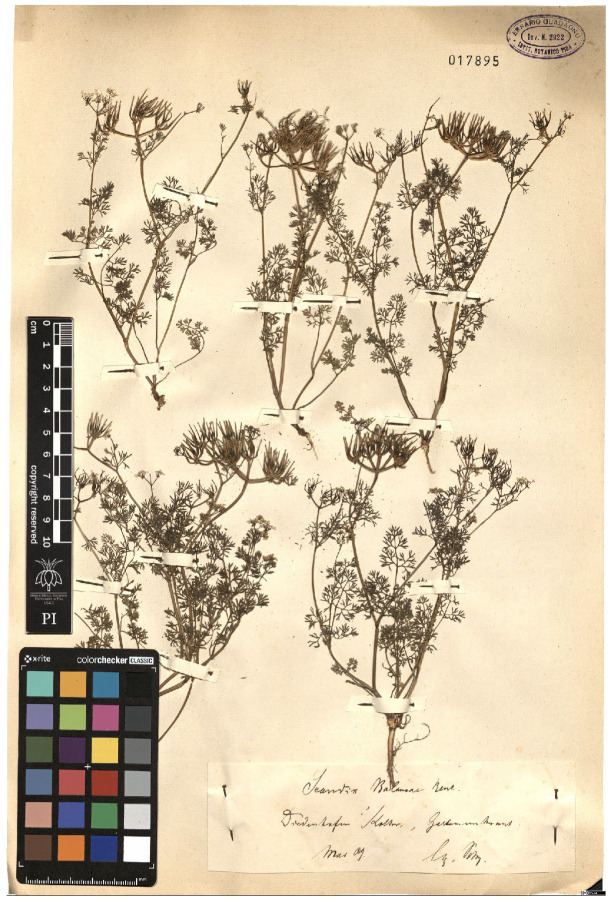	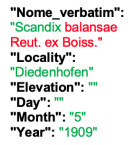	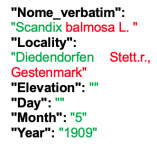	0.231

**Table 5:** Recognition examples with high TED accuracy from the fine-tuned model using 1200 × 1600 images. Labels containing only minor additional information or special characters, as well as images with tidy handwriting, generally yield high TED accuracy scores. Text shown in red indicates discrepancies between the ground truth and the prediction.

Specimen (Figure A5 and Figure A6)	Ground Truth	Recognition	Accuracy Score
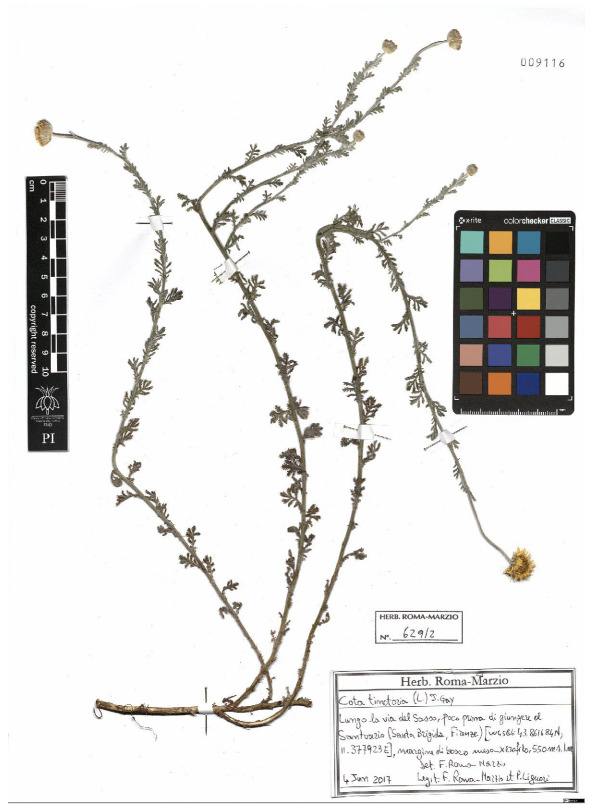	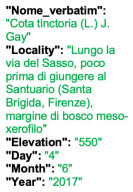	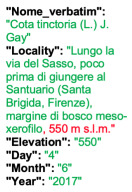	0.899
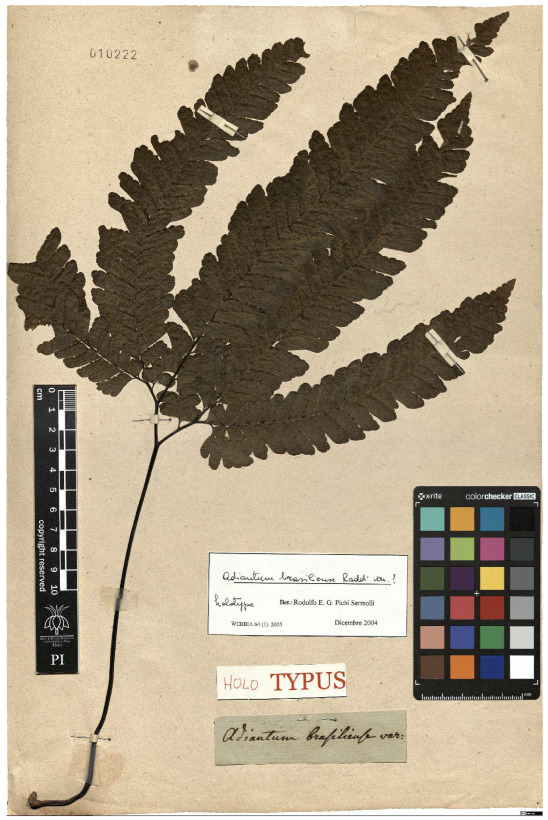	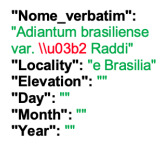	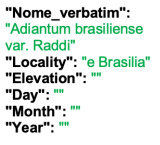	0.844

**Table 6:** Recognition examples with TED accuracy score of 1 from the fine-tuned model using 1200 × 1600 images. Specimen sheets with a single label, little to no additional information and clean handwritten or typewritten text typically achieve high TED accuracy scores.

Specimen (Figure A7 and Figure A8)	Ground Truth	Recognition	Accuracy Score
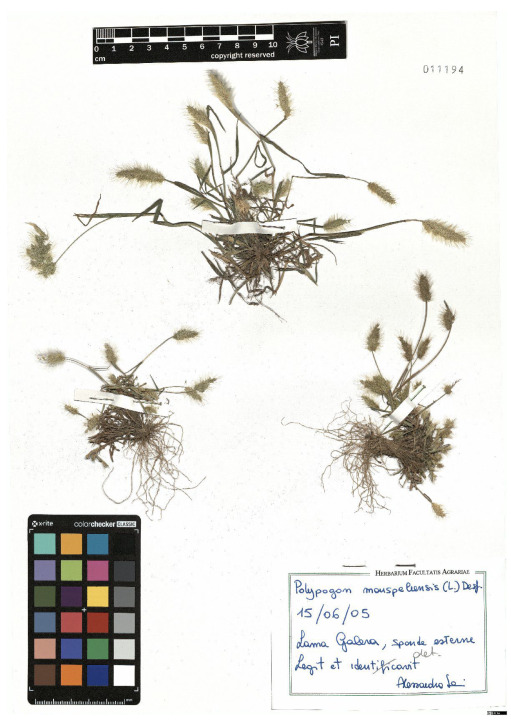	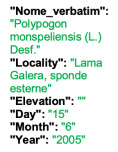	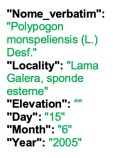	1.0
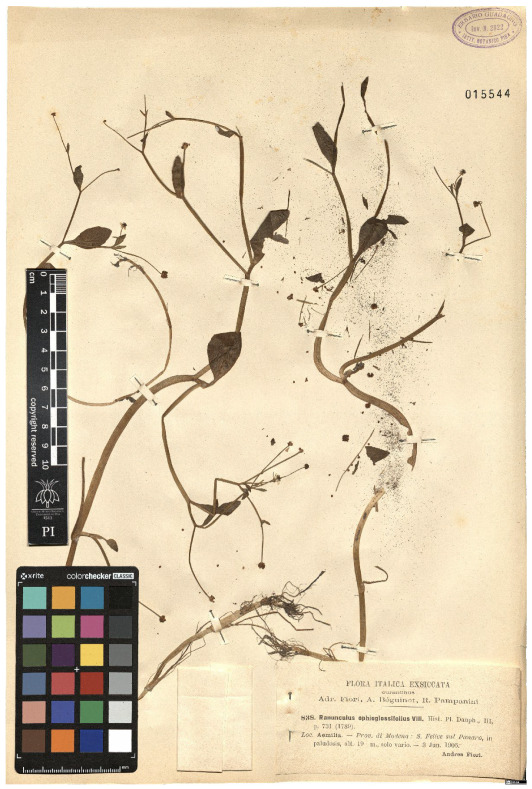	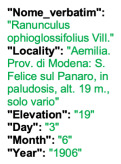	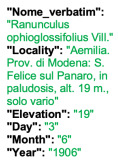	1.0

**Table 7:** Comparison of the capabilities of different base models performed using the widely adopted CORD benchmark dataset. Four features were selected for evaluating the models: (1) Text recognition: the OCR should not be a bottleneck for text extraction; (2) Multi-lingual support: the base model should be able to handle text in multiple languages; (3) Complex format support: the model should accurately recognize text across a variety of document formats; (4) CORD score: the Tree Edit Distance (TED) accuracy in CORD benchmark. Texts in bold indicate the match with our preference in the base model selection.

	(1) Text Recognition	(2) Multi-Lingual Support	(3) Complex Format Support	(4) CORD Score (Tree-Based Edit Distance Accuracy) [29]
BERT [35]	External OCR	**Yes**	No	65.5
BROS [36]	OCR	No	No	70.0
LayoutLM [37]	OCR	No	**Yes**	81.3
LayoutLM v2 [38]	OCR	**Yes**	**Yes**	82.4
TrOCR [28]	**OCR-free**	No	No	NA
Donut [29]	**OCR-free**	**Yes**	**Yes**	**90.9**

**Table 8:** The batch size selected for the three fine-tuning variations. The batch size is constrained by the 64 GB GPU memory and is depends on the storage size of each image. Consequently, higher-resolution images require smaller the batch sizes.

	600 × 800	960 × 1280	1200 × 1600
Batch size	10	8	5

## Data Availability

The original data presented in the study are openly available in GBIF at https://doi.org/10.15468/soyil7, accessed on 12 February 2026.
